# Cytotoxic Indole Diterpenoids from a *Sphagneticola trilobata*-Derived Fungus *Aspergillus* sp. PQJ-1

**DOI:** 10.3390/molecules28207003

**Published:** 2023-10-10

**Authors:** Wenxing Li, Guohui Yi, Kaiwen Lin, Guangying Chen, Yang Hui, Wenhao Chen

**Affiliations:** 1 Key Laboratory of Tropical Medicinal Resource Chemistry of Ministry of Education, College of Chemistry and Chemical Engineering, Hainan Normal University, Haikou 571158, China; wenxing2256@163.com (W.L.); chgying123@163.com (G.C.); 2 Key Laboratory of Tropical Medicinal Plant Chemistry of Hainan Province, College of Chemistry and Chemical Engineering, Hainan Normal University, Haikou 571158, China; 3 Public Research Center, Hainan Medical University, Haikou 571199, China; guohuiyi6@hainmc.edu.cn; 4 Hainan Women and Children’s Medical Center, Haikou 571158, China; kevinlinkaiwen@163.com

**Keywords:** *Sphagneticola trilobata*, *Aspergillus* sp. PQJ-1, secondary metabolites, cytotoxic activity

## Abstract

Two new indole diterpene derivatives, 5*S*-hydroxy-*β*-aflatrem (**1**) and 14*R*-hydroxy-*β*-aflatrem (**2**), along with one known analogue, 14-(*N*,*N*-dimethl-*L*-valyloxy)paspalinine (**3**), were isolated from the fermentation broth of the fungus *Aspergillus* sp. PQJ-1 derived from *Sphagneticola trilobata*. The structures of the new compounds were elucidated from spectroscopic data and ECD spectroscopic analyses. All the compounds (**1**–**3**) were evaluated for their cytotoxicity against A549, Hela, Hep G2, and MCF-7 cell lines. Compounds **1** and **2** exhibited selective inhibition against Hela cells. Further studies showed that **1** significantly induced apoptosis and suppressed migration and invasion in Hela cells. Moreover, **1** could up-regulate pro-apoptotic genes BAX and Caspase-3 and down-regulate anti-apoptotic genes Bcl-xL and XIXP.

## 1. Introduction

Indole diterpenes are a large, structurally diverse group of fungal secondary metabolites featuring an indole moiety fused to a diterpene skeleton and have been demonstrated to be an important source of structurally new and biologically active indole alkaloids [1,2,3]. These indole diterpenes have been mainly obtained from nine genera: including *Aspergillus*, *Chaunopycnis*, *Cladosporium*, *Dichotomomyces*, *Drechmeria*, *Eupenicillium*, *Mucor*, *Penicillium*, and *Tolypocladium* [4,5,6]. Indole diterpenoids have attracted extensive attention for their diverse skeletons [7,8,9] as well as their different biological activities, such as cytotoxicity [10] and anti-insectant [7], antifungal [11], antibiotic [12,13], anti-H1N1 [14], and tremorgenic activities [15]. The paxilline-type indole diterpenoids are the largest group of indole diterpenoids [16], and they feature an indole ring fused to a tetracyclic diterpene, many of which are biologically active [17,18,19].

**Figure 1:**
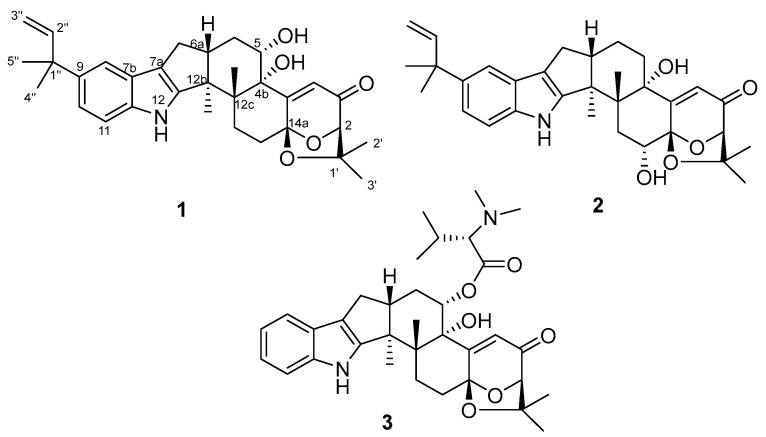
The structures of compounds **1**–**3**.

As part of our search for biologically active secondary metabolites from fungi of plant origin [20,21], we investigated *Aspergillus* PQJ-1, isolated from *Sphagneticola trilobata*. Subsequent chemical studies on EtOAc extract of the culture of this fungal strain led to the isolation of two new paxilline-type indole diterpene derivatives, 5*S*-hydroxy-*β*-aflatrem (**1**) and 14*R*-hydroxy-*β*-aflatrem (**2**), along with one known analogue, 14-(*N,N*-dimethl-*L*-valyloxy)paspalinine (**3**) (Figure 1). Compounds **1** and **2** displayed better inhibitory activity against Hela cells. By testing the relevant apoptosis genes, it can be tentatively deduced that **1** led to cell death by inducing apoptosis. Moreover, **1** could decrease Hela cell migration and invasion.

## 2. Results and Discussion

### 2.1. Structural Identification of Compounds ***1***–***2***

**Figure 2:**
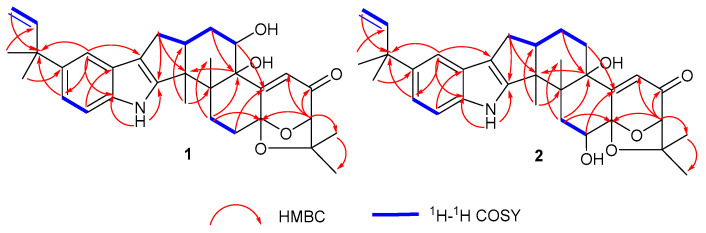
^1^H-^1^H COSY and key HMBC correlations for compounds **1**–**2**.

Compound **1** was obtained as a white amorphous powder. The HRESIMS showed the [M-H]^−^ at *m*/*z* 516.2753 to be consistent with the molecular formula C_32_H_39_NO_5_, accounting for 14 degrees of unsaturation (Figure S8). The characteristic absorption at λ_max_ (logε) 253 (1.55) and 283 (1.73) nm from the UV spectra indicated that the compound contained an indole structural fragment [22]. The ^1^H NMR spectrum (Figure S1) showed a characteristic indole hydrogen signal at *δ*_H_ 10.64 (1H, s, 12-NH); multiple overlapping methylene hydrogen signals between *δ*_H_ 1.84–2.86; and six methyl signals—*δ*_H_ 1.44 (3H, s, H-5″), 1.43 (3H, s, H-4″), 1.37 (3H, s, H-2′), 1.27 (3H, s, Me-12b), 1.13 (3H, s, Me-12c), and 1.08 (3H, s, H-3′) (Table 1). The above hydrogen spectral signals suggested that the compound possessed the indole diterpenoid skeleton [23]. The ^13^C NMR spectrum (Figure S2) combined with the DEPT spectrum (Figure S3) revealed the presence of 32 carbon signals in the compound, including one carbonyl carbon signal, *δ*_C_ 197.2; six alkenyl carbon signals, *δ*_C_ 167.7 (C-4a), 151.4 (C-12a), 149.2 (C-2″), 111.4 (C-3″), 119.8 (C-4), and 114.6 (C-7a); and six methyl carbon signals, *δ*_C_ 29.4 (C-5″), 29.2 (C-4″), 28.4 (C-2′), 22.9 (C-3′), 22.4 (Me-12c), and 16.0 (Me-12b). Comparison of the NMR spectra of this compound with Compound **3** revealed that the substituent C_7_H_4_NO was missing at position 5 in compound **1**, and the carbon signal at C-5 in **3** was shifted from *δ*_C_ 74.8 in high field to *δ*_C_ 69.8 in **1**. It was thus speculated that a hydroxyl substituted at C-5, which was further confirmed by the correlation signals in the ^1^H-^1^H COSY spectrum (Figure S5) between *δ*_H_ 5.24 (1H, s, OH-5) and *δ*_H_ 4.10 (H, m, H-5) and the correlation signals between OH-5 and C-4b/6 in the HMBC spectrum (Figure S6). Furthermore, correlations between H-4 and C-4b/2/14a can also be observed in the HMBC spectrum. Compound **1** had an additional C_5_H_9_ substituent at C-9 compared to **3**, as confirmed by the correlation signals in the HMBC spectra of H-5″ with C-1″/2″, H-4″ with C-1″/9, and H-8/10 with C-1″ (Figure 2). The planar structure of **1** was thus determined.

**Figure 3:**
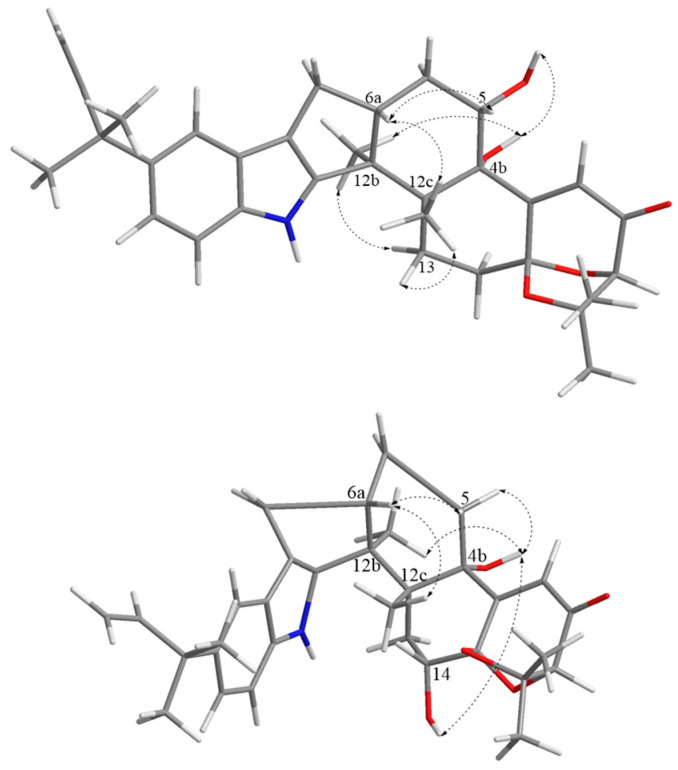
Key NOESY correlations for compounds **1**–**2** (Blue for nitrogen atoms, red for oxygen atoms).

**Figure 4:**
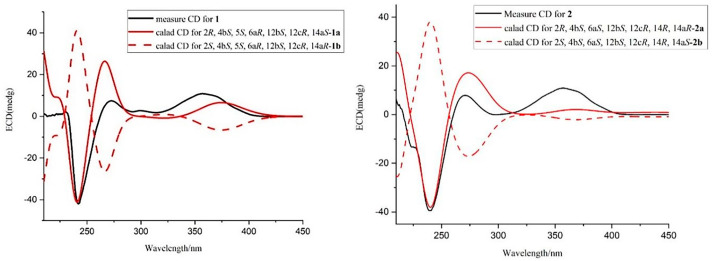
Experimental CD spectra and the calculated ECD spectra of Compounds **1**–**2**.

The key NOESY correlations in the NOESY spectra (Figure S7) of Me-12b (*δ*_H_ 1.27) with H-13*α* (*δ*_H_ 2.44) and OH-4b (*δ*_H_ 4.52) and Me-12c (*δ*_H_ 1.13) with H-6a (*δ*_H_ 2.67), H-5 (*δ*_H_ 4.10), and H-13*β* (*δ*_H_ 2.72) (Figure 3) indicated that Me-12b, H-13*α,* and OH-4b were in the same direction, while Me-12c, H-13*β*, H-5, and H-6a were in the same direction. Furthermore, previous studies had shown that the strongest negative cotton effect at short ECD wavelengths (λ_max_ 210–250 nm) came from the π-π* leaps of the indole nucleus, which could be used to determine the absolute configurations of C-4b, C-6b, C-12b, and C-12c in hexacyclic indole diterpenoids [24]. Thus, the absolute configurations of 4b, 5, 6a, 12b, 12c could be determined as 4b*S*, 5*S*, 6a*R*, 12b*S*, and 12c*R* based on λ_max_ 242 (Δε −188.7) nm in CD and the associated signals in NOESY. While the absolute configurations at positions 2 and 14a were determined by comparing the carbon signals with the calculated values of the NMR data from the literature [24], it was hypothesized that there were two possible configurations, **1a** (2*R*, 4b*S*, 5*S*, 6a*R*, 12b*S*, 12c*R*, 14a*S*) and **1b** (2*S*, 4b*S*, 5*S*, 6a*R*, 12b*S*, 12c*R*, 14a*R*), for compound **1**. ECD calculations of the two configurations were carried out using Boltzmann-weighted time-varying density flooding theory (TDDFT) [25]. Geometry optimization was carried out using the DFT method, and the geometry-optimized conformations were further calculated at the B3LYP/6-311G* level using SMD [26]. The calculated values were compared with the experimental values, and it was found that the ECD calculated values of **1a** coincided with the experimental values (Figure 4), from which the absolute configuration of **1** was determined as 2*R*, 4b*S*, 5*S*, 6a*R*, 12b*S*, 12c*R*, 14a*S*, and it was designated as 5*S*-hydroxy-*β*-aflatrem.

Compound **2** was obtained as a white amorphous powder. Its molecular formula was determined to be C_32_H_39_NO_5_ based on a HRESIMS peak at *m*/*z* 516.2753 [M-H]^−^ (Figure S16), and the degree of unsaturation was 14, which was consistent with the molecular formula and degree of unsaturation of **1**. Comparison of the NMR data of **2** with those of **1** revealed that the NMR data of the two compounds were similar (Table 1), but from the correlation signals of H-13 with C-14a and OH-14 with C-13/14a in the HMBC spectrum (Figure S14) combined with the correlation signals of *δ*_H_ 1.86 (2H, m, H-13) and *δ*_H_ 4.10 (1H, t, *J* = 6.4 Hz, H-14) in ^1^H-^1^H COSY spectrum (Figure S13), it could be inferred that the hydroxyl substitution in compound **2** was at C-14 (Figure 2), and thus, the planar structure of compound **2** could be determined.

In the NOESY spectra (Figure S15), the key NOESY correlations of Me-12b (*δ*_H_ 1.28) with H-5*α* (*δ*_H_ 2.75) and OH-4b (*δ*_H_ 4.54) and Me-12c (*δ*_H_ 1.08) with H-6a (*δ*_H_ 2.73), H-14, and H-5*β* (*δ*_H_ 1.83) (Figure 3) showed that Me-12b, H-5*α*, OH-4b, and OH-14 were in the same direction, while Me-12c, H-5*β*, H-14, and H-6a were in the same direction. The absolute configurations of 4b, 6a, 12b, 12c, and 14 could be identified as 4b*S*, 6a*S*, 12b*S*, 12c*R*, and 14*R*, respectively, based on the negative cotton effect at λ_max_ 239 (Δε − 90.7) nm in CD [24]. Comparing the carbon signals at positions 2 and 14a with the calculated values from the NMR data in the literature [24], it was hypothesized that there were two possible configurations, **2a** (2*R*, 4b*S*, 6a*S*, 12b*S*, 12c*R*, 14*R*, 14a*R*) and **2b** (2*S*, 4b*S*, 6a*S*, 12b*S*, 12c*R*, 14*R*, 14a*S*) for compound **2**. The ECD calculations of **2a** and **2b** were performed using TDDFT [25], and the calculated values were compared with the experimental values of **2**. It was found that the ECD calculated values of **2a** were in better agreement with the experimental values (Figure 4), which allowed us to determine the absolute configuration of **2** as 2*R*, 4b*S*, 6a*S*, 12b*S*, 12c*R*, 14*R*, 14a*R* and to name it as 14*R*-hydroxy-*β*-aflatrem.

### 2.2. Cytotoxic Activity of Compounds ***1***–***3***

Compounds (**1**–**3**) were evaluated for their cytotoxicity against four cancer cell lines, including lung cancer cell A549, cervical cancer cell Hela, liver cancer cell Hep G2, and breast cancer cell MCF-7 with various concentrations for 48 h and then performed methyl thiazol tetrazolium (MTT) assays. Compound **1** displayed better inhibitory activities against Hela, Hep G2, and MCF-7 cells, with IC_50_ values of 12.54, 15.06, and 26.56 µM, respectively (Table 2). The most active, Compound **1**, was selected for further research.

### 2.3. Compound ***1*** Could Cause Apoptosis of Hela Cell

**Figure 5:**
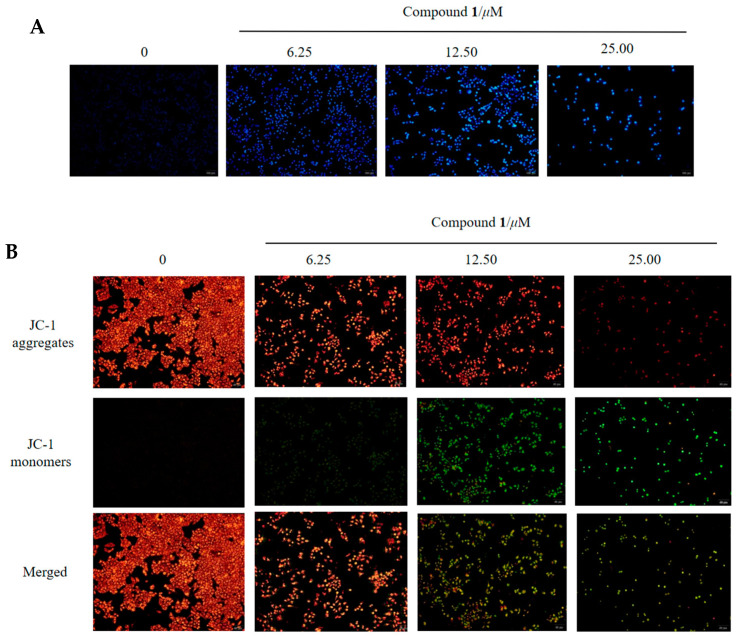

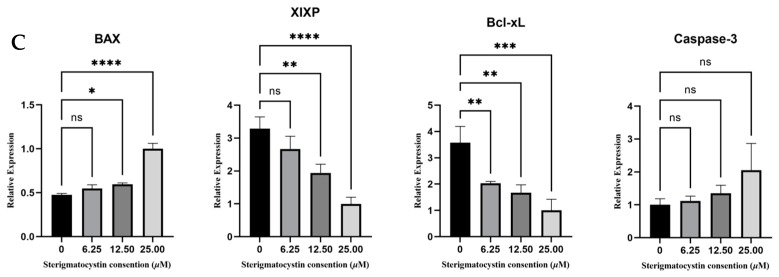
Apoptosis and mitochondrial membrane potential were analyzed via real-time quantitative fluorescence PCR and fluorescence microscopy in Hela cells treated with **1** for 48 h. (**A**) Hoechst 33258 staining showed that **1** could induce nuclear shrinkage in Hela cells. (**B**) Reduction in mitochondrial membrane potential in Hela cells after treatment with **1** for 48 h via JC-1 staining assays. (**C**) Compound **1** induced changes in the mRNA levels of related genes in Hela cells after treatment for 48 h at 0, 6.25, 12.50, and 25.00 µM. Values were shown as the means ± standard deviation; * *p* < 0.05, ** *p* < 0.01, *** *p* < 0.005, **** *p* < 0.001, ns (no significance) compared with the control group.

Apoptosis, which occurs normally during development and aging, is a homeostatic mechanism for maintaining tissue cell populations and an important strategy for treating cancer [27]. Apoptosis leads to cell death with cell nuclear pyknosis and apoptotic body production, which was observed via fluorescence microscopy after Hoechst 33258 staining [27,28]. As shown in Figure 5A, untreated cells showed uniform blue fluorescence, but Hela cells treated with **1** for 48 h showed clear apoptotic signals, with shrinkage of chromatin, fragmentation of nuclei, and bright blue fluorescence, and apoptotic bodies appeared in apoptotic cells.

We further investigated the effect of **1** on apoptosis using the mitochondrial membrane potential assay kit (JC-1) [29]. Decrease in mitochondrial membrane potential was a hallmark event in the early stage of apoptosis. The decrease in membrane potential could be easily detected by the shift of JC-1 from red to green fluorescence, and the shift of JC-1 from red to green fluorescence could also be used as an indicator of the early stage of cell apoptosis. Treatment of Hela cells with 0, 6.25, 12.50, and 25.00 µM concentrations of **1** for 48 h showed a decrease in mitochondrial membrane potential with increasing concentration (Figure 5B). Further analysis of the expression of proapoptotic and anti-apoptotic genes via real-time fluorescence quantitative PCR that **1** could up-regulate pro-apoptotic genes BAX and Caspase-3 but down-regulate anti-apoptotic genes Bcl-xL and XIXP (Figure 5C). These results indicated that compound **1** promoted cell apoptosis.

### 2.4. Wound Healing and Transwell Assay

**Figure 6:**
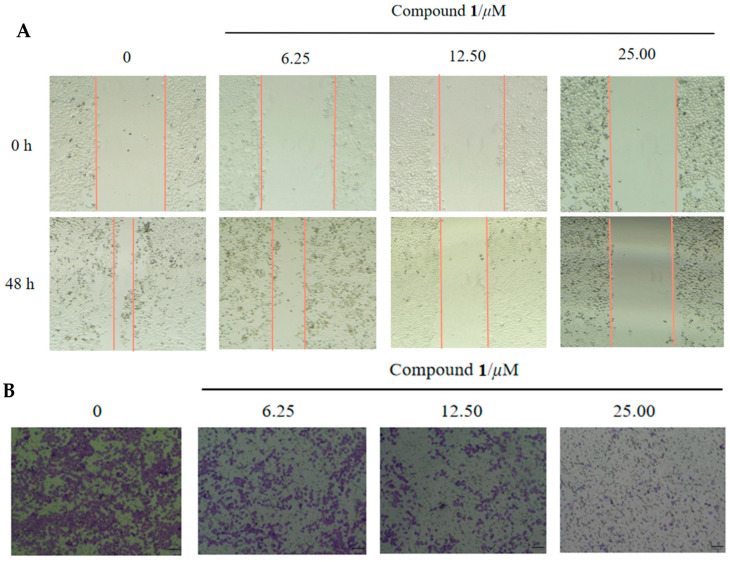
(**A**) Wound healing assay to determine migrating potentials of Hela cells. The area alteration of the wound after treatment with compound **1** for 48 h showed the migration ability of cells. (**B**) Transwell assays to determine invasion abilities of Hela cells.

Wound healing is one of the hallmarks of tumorigenesis. As shown in Figure 6A, treatment of cells with compound **1** for 48 h significantly inhibited the migration ability of Hela cells. In line with the wound-healing assay, a transwell assay displayed that the level of cell invasion was strikingly decreased after 48 h treatment with **1**, although cell viability was unaffected (Figure 6B) [30]. These results indicated that **1** was also able to antagonize cell migration and invasion in the Hela cells, with potential activity to inhibit the metastasis of tumor cells.

## 3. Materials and Methods

### 3.1. General Experimental Procedures

Optical rotation spectra were recorded on a Modular polarimeter MCP 5100 (JASCO, Tokyo, Japan). ECD spectra and UV spectra were measured using a Biologic MOS450-SFM300 instrument (JASCO, Tokyo, Japan). The Spartan 14 program (Wavefunction Inc., Irvine, CA, USA) was used for calculating Merck molecular force field (MMFF). The Gaussian 16 program package1 was used for density functional theory (DFT) and time-dependent density functional theory (TDDFT) calculations. NMR spectra were recorded on a Bruker AV spectrometer (400 MHz for ^1^H NMR and 100 MHz for ^13^C NMR) (Bruker Corporation, Switzerland) instrument using DMSO-*d*_6_ as a solvent. Tetramethyl silane (TMS) was used as an internal standard. HRESIMS spectrometer were measured on a Q-TOF Ultima Global GAA076 LC mass spectrometer. (Billerica, MA, USA). Semi-Preparative HPLC was performed on an Agilent 1260 LC series with a DAD detector using an Agilent Eclipse XDBC18 column (9.4 250 mm, 5 µm) (Agilent Corporation, Santa Clara, CA, USA). Silica gel (Qing Dao Hai Yang Chemical Group Co.; 200–300 mesh) and octadecyl silyl silica gel (YMC; 12 nm–50 µm) were used for column chromatography (CC). Precoated silica gel plates (Yan Tai Zi Fu Chemical Group Co. (Yantai, China); G60, F-254) were used for thin-layer chromatography (TLC).

### 3.2. Fungal Materials

The endophytic fungal strain *Aspergillus* sp. PQJ-1 was isolated from *Sphagneticola trilobata* harvested from Hainan Normal University of Hainan Province, China, in August 2020. *S, trilobata* was identified by Prof. Qiong-Xin Zhong, College of Life Science, Hainan Normal University. The strain was identified as *Aspergillus* sp. on the basis of the morphological, physiological, and biochemical characteristics and DNA sequencing of the ITS region of the rRNA gene, and the sequence of *Aspergillus* sp. submitted to GenBank with accession number OM269036. The endophytic fungus *Aspergillus* sp. PQJ-1 had been preserved at the Key Laboratory of Tropical Medicinal Resource Chemistry of Ministry of Education, College of Chemistry and Chemical Engineering, Hainan Normal University, Haikou, Hainan, China.

### 3.3. Fermentation, Extraction, and Isolation

The fungal strain *Aspergillus* sp. PQJ-1 was cultured in potato liquid medium PDB at 28 °C without shaking for 35 days. The fermented cultures of *Aspergillus* sp. PQJ-1 were extracted 3 times with EtOAc, and the solvents were evaporated under reduced pressure to obtain an extract (150.0 g).

The total crude extract was subjected to silica gel column chromatography (CC) and eluted with chloroform/acetone (10:1–0:10, *v*/*v*) to give seven fractions (Fr.1–Fr.7). Fr.2 was further separated on an ODS column using a stepped gradient elution of MeOH-H_2_O (10:90–100:0, *v*/*v*) to yield five subfractions (Fr.2.1–Fr.2.5). Fr.2.3 was further separated on a Sephadex LH-20 column with chloroform/MeOH (1:1, *v*/*v*) to provide four subfractions (Fr.2.3.1–Fr.2.3.4). Fr.2.3.2 was further separated using HPLC eluted with CH_3_CN/H_2_O (70:30, *v*/*v*) to obtain Compounds **1** (21 mg), **2** (17 mg), and **3** (6.7 mg), respectively.

5*S*-hydroxy-*β*-aflatrem (**1**): white amorphous powder; ${\left[\alpha \right]}_{D}^{20}$ = +111.6 (*c* 0.077, MeOH); UV (MeOH) *λ*_max_ [log *ε*/(L·mol^−1^·cm^−1^)]: 253 (1.55), 284 (1.73) nm; HR-ESI-MS *m*/*z* 516.2753 [M − H]^−^ (calcd for C_32_H_38_NO_5_, 516.2728). ^1^H and ^13^C NMR data are in Table 1.

14*R*-hydroxy-*β*-aflatrem (**2**): white amorphous powder; ${\left[\alpha \right]}_{D}^{20}$ = +50.0 (*c* 0.050, MeOH); UV (MeOH) *λ*_max_ [log *ε*/(L·mol^−1^·cm^−1^)]: 242 (1.39), 285 (0.54) nm; HR-ESI-MS *m*/*z* 516.2756 [M − H]^−^ (calcd for C_32_H_38_NO_5_, 516.2728). ^1^H and ^13^C NMR data are in Table 1.

### 3.4. Cytotoxicity Assays

The cytotoxic activities of **1**–**3** against human lung cancer cells A549, cervical cancer cells Hela, hepatocellular carcinoma cells Hep G2, and breast cancer cells MCF-7 were evaluated via MTT assay using Adriamycin (ADM) as a positive control [31,32]. The cells in were taken in the logarithmic growth phase and inoculated with 96-well plate (1 × 10^5^ cells/well), incubated in the incubator at 37 °C overnight, and then treated with various concentrations of drug for 48 h. They were then incubated in the constant-temperature carbon dioxide incubator for 48 h and then taken out the 96-well plate. Following this, 10 µL of pre-thawed MTT at room temperature was added to each well and placed into the incubator for 4 h. After being taken out the 96-well plate, the supernatant was poured off, and the extra liquid was removed and blotted on a paper towel. Then, 150 µL of DMSO was added to each well and shaken away from light to dissolve the dirty purple crystals. The plate cover was removed, and the absorbance values at 570 nm were determined using an enzyme counter. The half-maximal inhibitory concentrations were determined using the GraphPad Prism 9 software.

#### 3.4.1. Cell Line and Cell Culture

Human cell lines A549, Hela, Hep G2, and MCF-7 were provided by the Hainan Normal University (Haikou, China). A549 cells were cultured in F12K medium (Pricella, Wuhan, China) supplemented with 10% fetal bovine serum (FBS) (Pricella). Hela, Hep G2, and MCF-7 cells were cultured in DMEM medium (Pricella) supplemented with 10% FBS (Pricella). All cells above were maintained in 5% CO_2_ at 37 °C. Cytotoxicity was measured using the MTT (Beyotime, Shanghai, China) assay.

#### 3.4.2. Wound-Healing Migration Assay

The cells were seeded in a six-well plate (4 × 10^6^ cells/well). When the cells had grown to a monolayer spread across the bottom of the well plate, a 10 µL pipette tip was used to make a wound in each well. After washing away the detached cells and debris with PBS, different concentrations of **1** were added and then incubated in 1% serum medium for 48 h [29]. The scratches were photographed at 0 and 48 h and assessed for migratory capacity.

#### 3.4.3. Transwell Migration Assay

After treating the Hela cells with different concentrations of Compound **1** for 48 h, the cells were digested with trypsin and resuspended in serum-free medium, with 200 µL of cells (5 × 10^4^) added to each of the upper chambers and 600 µL of medium containing 10% serum added to the lower chambers to be incubated at 37 °C for 48 h. The small chambers were removed, the medium was aspirated, the cells in the upper chamber were gently wiped with a cotton swab. The chambers were fixed in 4% paraformaldehyde for 20 min and air-dried, followed by staining with 0.1% crystal violet stain for 10 min and washing with PBS 3 times. Cell counts were obtained by observing the membranes under a microscope, counting the cells in each field [30].

#### 3.4.4. Quantitative Real-Time Polymerase Chain Reaction (qPCR) Analysis

The cells after 48 h of treatment with compound **1** were digested with trypsin, centrifuged and the supernatant discarded, 200 µL of RNA lysate was added and the cells were crushed on ice. The total RNA was extracted using a total RNA isolation kit (Puluomaige Biotech Co. Ltd., Beijing, China). The isolated total RNA was reverse-transcribed to cDNA using the Hifair Ⅲ 1st Strand cDNA Synthesis SuperMix (gDNA digester plus) kit (Yeasen Biotechnology Co., Ltd., Shanghai, China). The mRNA levels were normalized to GAPDH expression in human tumor cells and analyzed relative to controls using the 2^−ΔΔCt^ method [33]. The qPCR reactions were performed with the following primers: Bcl-xL-F: 5′-TGCGTGGAAAGCGTAGACAAG-3′; Bcl-xL-R: 5′-GTGGGAGGYAGAGTGGATGG-3′; BAX-F: 5′-TTTTGCTTCAGGGTTTCA-3′; BAX-R: 5′-GGACATCAGTCGCTTCAGT-3′; Caspase-3-F: 5′-ATTGATGCGTGATGTT-3′; Caspase-3-R: 5′-CAGGTGCTGTGGAGTA-3′; XIXP-F: 5′-TAGTGCCACGCAGTCT-3′; XIXP-R: 5′-AGGGTTCCTCGGGTAT-3′; GAPDH-F: 5′-AATCCCATCACCATCTTC-3′; GAPDH-R: 5′-TCACGCCACAGTTTCC-3′.

#### 3.4.5. Mitochondrial Membrane Assay

Cells were seeded in six well plates (1 × 10^4^ cell/well). When the cells were walled and incubated for 48 h with different concentrations of the drug. The culture solution was aspirated and incubated with JC-1 staining solution for 20 min at 37 °C [28]. The supernatant was then aspirated and washed twice with JC-1 staining buffer before being observed under a fluorescence microscope and photographed.

#### 3.4.6. Observation of Morphological Changes in Hela Cells by Hoechst 33258 Staining

Hela cells were inoculated in 24-well plates at 1 × 10^5^ cells/well, and after walling, the cells were treated with compound **1** at concentrations of 0, 6.25, 12.50, 25.00 µM, respectively, and cultured for 48 h in an incubator at 37 °C [28]. The cells were washed twice with PBS, stained with Hoechst 33258 stain for 15 min, washed twice with PBS, and then photoprocessed with an OLYMPUS IX-51 fluorescence microscope.

## 4. Conclusions

In a summary, this study disclosed two previously undescribed paxilline-type indole diterpene derivatives, named 5*S*-hydroxy-*β*-aflatrem (**1**) and 14*R*-hydroxy-*β*-aflatrem (**2**), along with one known analogue, 14-(*N*,*N*-dimethl-*L*-valyloxy)paspalinine (**3**), which were isolated from the endophytic fungus *Aspergillus* sp. PQJ-1, derived from the flowers of *S. trilobata*. The structures of the new compounds with absolute configurations were determined unambiguously by NMR spectroscopic data analysis and quantum chemical calculations. Compounds **1**–**2** possessed cytotoxic activity. The investigation of the underlying mode of action confirmed that compound **1** might exert antitumor activity through a decrease in mitochondrial membrane potential, up-regulation of pro-apoptotic genes and down-regulation of anti-apoptotic genes, and inhibition of cell migration and invasion.

## Figures and Tables

**Table 1:** ^1^H (400 MHz) and ^13^C NMR (100 MHz) data for **1**–**2** (DMSO-*d*_6_).

Position	1		2	
	*δ*_C_	*δ*_H_ (*J* in Hz)	*δ*_C_	*δ*_H_ (*J* in Hz)
2	87.1	4.39, s	87.1	4.39, s
3	197.2		197.3	
4	119.8	6.24, br s	119.8	6.24, s
4a	167.7		167.8	
4b	78.8		77.6	
5	69.8	4.10, m	28.2	1.83, m 2.75, m
6	31.3	1.84, m	26.8	1.92, m 2.46, m
6a	44.8	2.67, m	45.4	2.73, m
7	26.6	1.95, m 2.49, m	26.6	2.32, m 2.56, m
7a	114.6		115.3	
7b	122.9		124.3	
8	115.6	6.85, d (1.2)	114.4	7.18, d (1.8)
9	138.8		138.4	
10	118.9	6.87, d (7.2)	118.2	6.94, dd (1.8, 9.0)
11	110.8	7.18, d (7.8)	111.4	7.19, d (8.6)
11a	140.8		138.2	
12		10.64, s		10.48, s
12a	151.4		152.4	
12b	49.9		50.6	
12c	39.6		39.6	
13	33.1	2.44, m 2.72, m	31.5	1.86, m
14	28.2	1.84, m 2.74, m	70.0	4.10, t (6.4)
14a	104.4		104.4	
12b-Me	16.0	1.27, s	16.2	1.28, s
12c-Me	22.4	1.13, s	22.9	1.08, s
1′	77.6		78.7	
2′	28.4	1.37, s	28.4	1.36, s
3′	22.9	1.08, s	22.4	1.13, s
1″	41.0		40.5	
2″	149.2	6.19, t (7.2)	149.0	6.03, q (6.4)
3″	111.4	4.81, dd (1.2, 10.8) 4.96, dd (1.2, 17.4)	109.8	5.00, t (10.4)
4″	29.2	1.43, s	28.6	1.37, s
5″	29.4	1.44, s	28.6	1.37, s
4b-OH		4.52, s		4.54, s
5-OH		5.24, s		
14-OH				5.29, s

s—sines-single; d—doublet; t—triplet; q—quartet; m—multiplet; br s—broad single; dd—doublet of doublets.

**Table 2:** Results of antitumor activities.

IC_50_ (µM)				
Compounds	A549	Hela	Hep G2	MCF-7
**1**	>50	12.54	15.06	26.56
**2**	>50	15.61	20.03	29.47
**3**	>50	>50	>50	>50
Adriamycin	5.52	1.50	5.73	5.24

## Data Availability

Not applicable.

## Supplementary Materials

The following supporting information can be downloaded at: https://www.mdpi.com/article/10.3390/molecules28207003/s1. Figures S1–S16: 1D, 2D NMR spectra and HR-ESI-MS of Compounds **1**–**2**.
