# Supplementary material for: Cytotoxic Indole Diterpenoids from a Sphagneticola trilobata-Derived Fungus Aspergillus sp. PQJ-1
Source: Molecules. 2023 Oct 10;28(20):7003. doi: 10.3390/molecules28207003 (PMC10609460; doi:10.3390/molecules28207003)
Supplement: Supplementary file 1 [file molecules-28-07003-s001.zip › molecules-2585845-supplementary.pdf]

SUPPLEMENTARY MATERIALS

Cytotoxic Indole-Diterpenoids from a *Sphagneticola trilobata* Derived Fungus *Aspergillus* sp. PQJ-1

Wenxing Li [1,2,], Guohui Yi [3,†], Kaiwen Lin [4], Guangying Chen [1,2], Yang Hui [1,2,*] and Wenhao Chen [1,2,*]

1 Key Laboratory of Tropical Medicinal Resource Chemistry of Ministry of Education, College of Chemistry and Chemical Engineering, Hainan Normal University, Haikou, 571158, China; wenxing2256@163.com (W.L.);

2 Key Laboratory of Tropical Medicinal Plant Chemistry of Hainan Province, Haikou, 571158, China;

3 Public Research Center Hainan Medical University, Haikou571199, China; guohuiyi6@hainmc.edu.cn (G.Y.);

4 Hainan Women and Children's Medical Center, Haikou, 571158, China; kevinlinkaiwen@163.com (K.L.);

* Correspondence: 070103@hainnu.edu.cn (W.C.); 070066@hainnu.edu.cn (Y.H.);

† These authors contributed equally to this work.

Abstract: Two new indole diterpene derivatives 5*S*-hydroxy-*β*-aflatrem (**1**), 14*R*-hydroxy-*β*-aflatrem (**2**), along with one known analogue 14-(*N,N*-dimethl-*L*-valyloxy)paspalinine (**3**), were isolated from the fermentation broth of the fungus *Aspergillus* sp. PQJ-1 derived from *Sphagneticola trilobata*. The structures of the new compounds were elucidated from spectroscopic data and ECD spectroscopic analyses. All the compounds (**1**-**3**) were evaluated for their cytotoxicity against A549, Hela, Hep G2, and MCF-7 cell lines. Compounds **1** and **2** exhibited selective inhibition against Hela cells. Further studies showed that compound **1** significantly induced apoptosis, and suppressed migration and invasion in Hela cells. Moreover, **1** could up-regulate pro-apoptotic genes BAX, Caspase-3, and down-regulate anti-apoptotic genes Bcl-xL, XIXP.

Keywords: *Sphagneticola trilobata*; *Aspergillus* sp. PQJ-1; secondary metabolites; cytotoxic activity

Contents

Figure S1. ^{1}H NMR spectrum of **1** in DMSO-d_6 3

Figure S2. ^{13}C NMR spectrum of **1** in DMSO-d_6 3

Figure S3. 135-DEPT spectrum of **1** in DMSO-d_6 4

Figure S4. HSQC spectrum of **1** in DMSO-d_6 4

Figure S5. ^{1}H-^{1}H COSY spectrum of **1** in DMSO-d_6 5

Figure S6. HMBC spectrum of **1** in DMSO-d_6 5

Figure S7. NOESY spectrum of **1** in DMSO-d_6 6

Figure S8. HRESIMS spectrum of **1** 6

Figure S9. ^{1}H NMR spectrum of **2** in DMSO-d_6 7

Figure S10. ^{13}C NMR spectrum of **2** in DMSO-d_6 7

Figure S11. 135-DEPT spectrum of **2** in DMSO-d_6 8

Figure S12. HSQC spectrum of **2** in DMSO-d_6 8

Figure S13. ^{1}H-^{1}H COSY spectrum of **2** in DMSO-d_6 9

Figure S14. HMBC spectrum of **2** in DMSO-d_6 9

Figure S15. NOESY spectrum of **2** in DMSO-d_6 10

Figure S16. HRESIMS spectrum of **2** 10

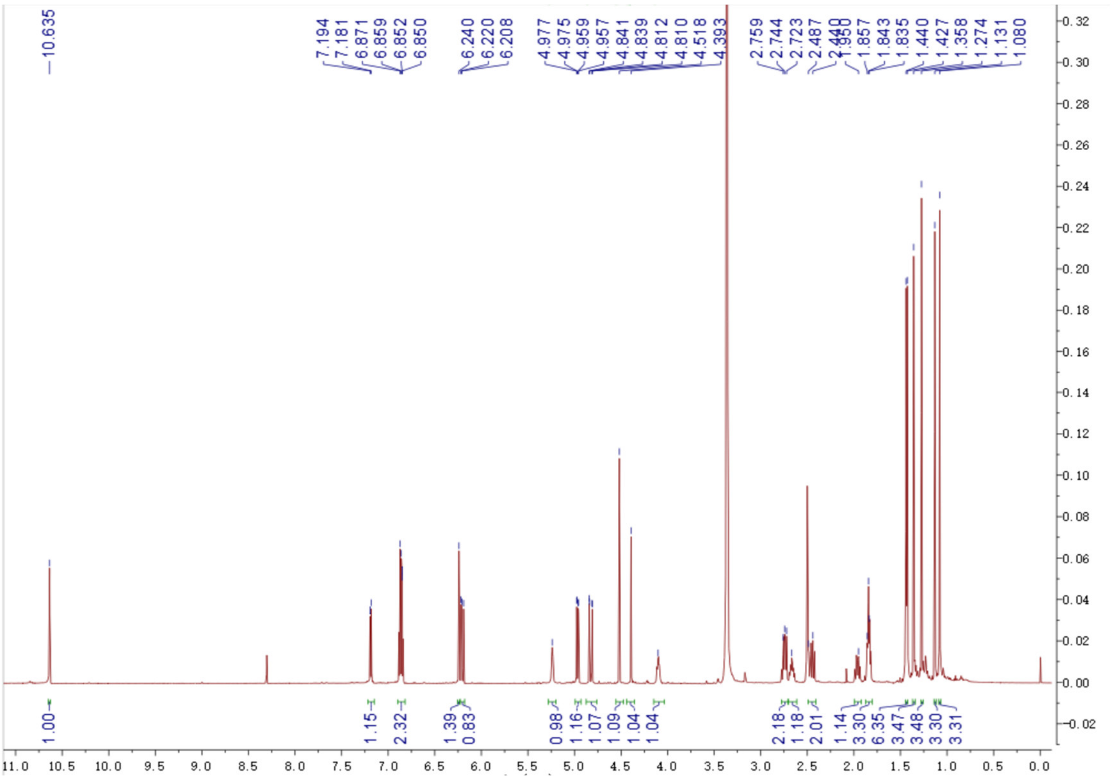

Figure S1. ^{1}H NMR spectrum of **1** in DMSO-d_6

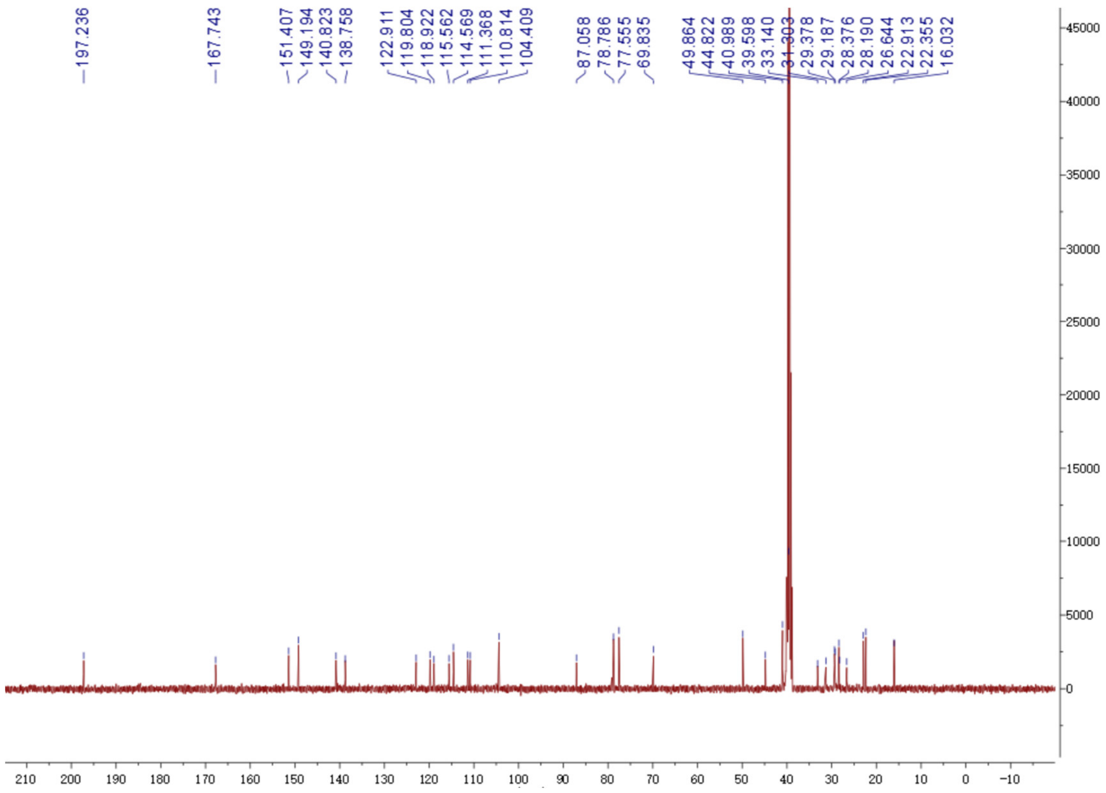

Figure S2. ^{13}C NMR spectrum of **1** in DMSO-d_6

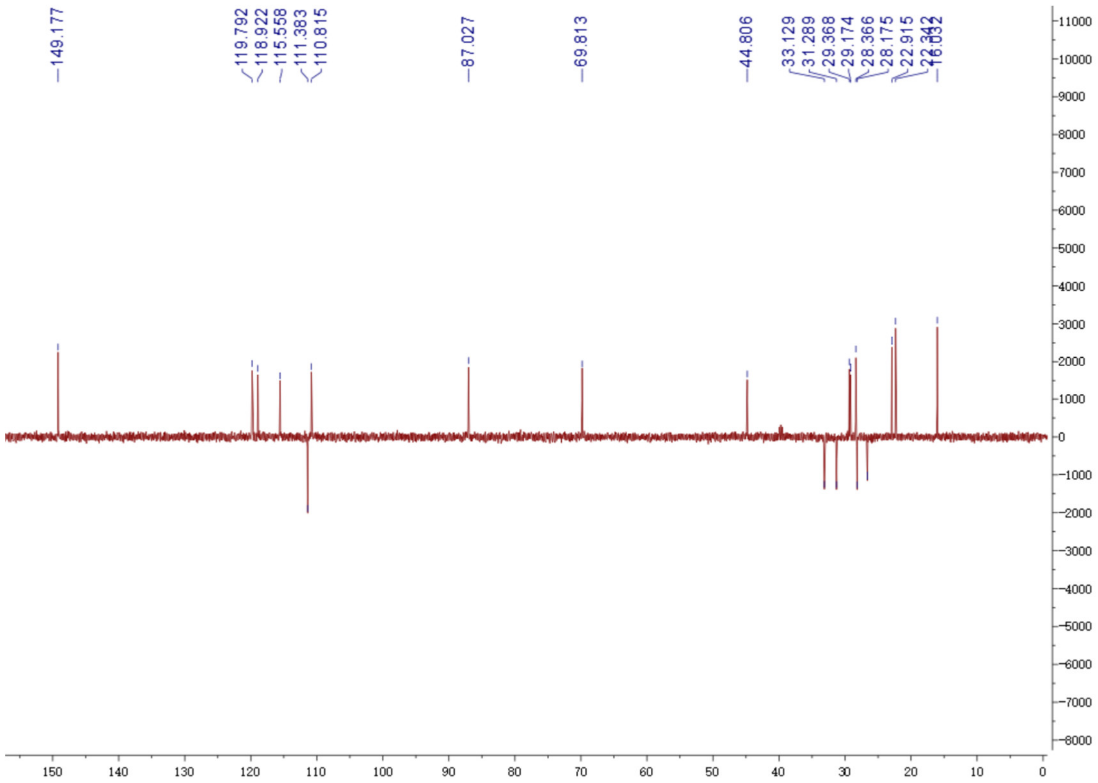

Figure S3. 135-DEPT spectrum of **1** in DMSO-d_6

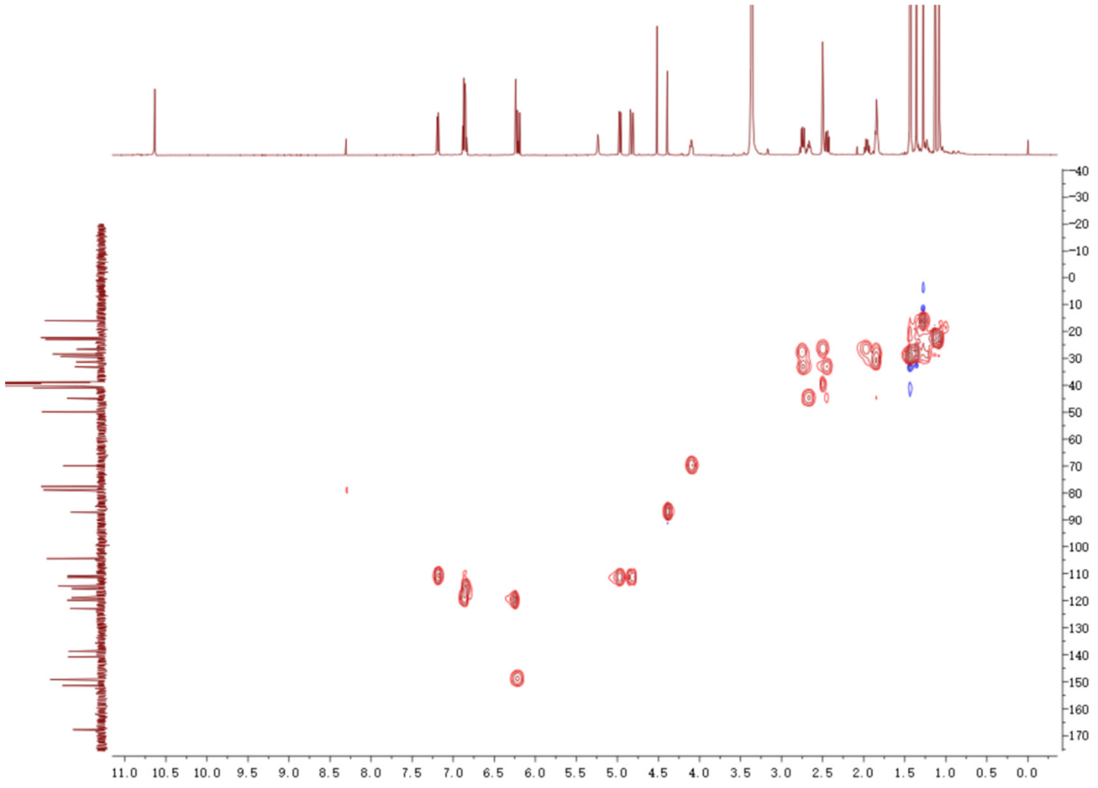
Figure S4. HSQC spectrum of **1** in DMSO-d_6

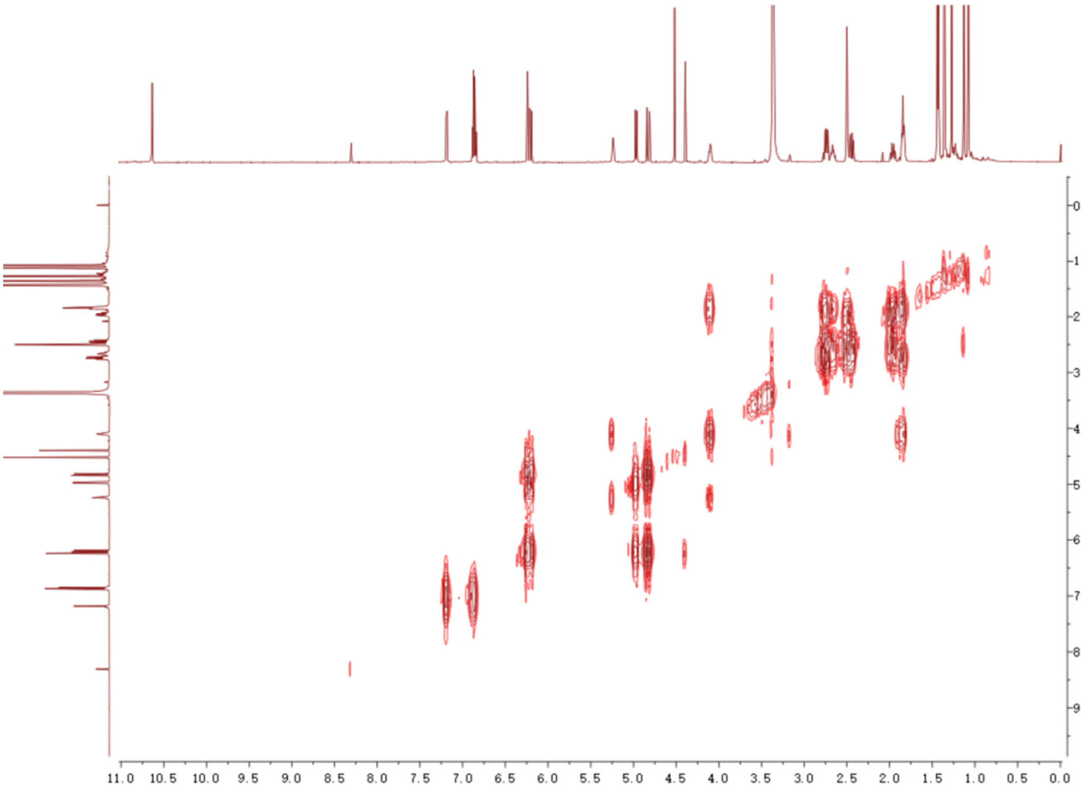

Figure S5. ^{1}H-^{1}H COSY spectrum of **1** in DMSO-d_6

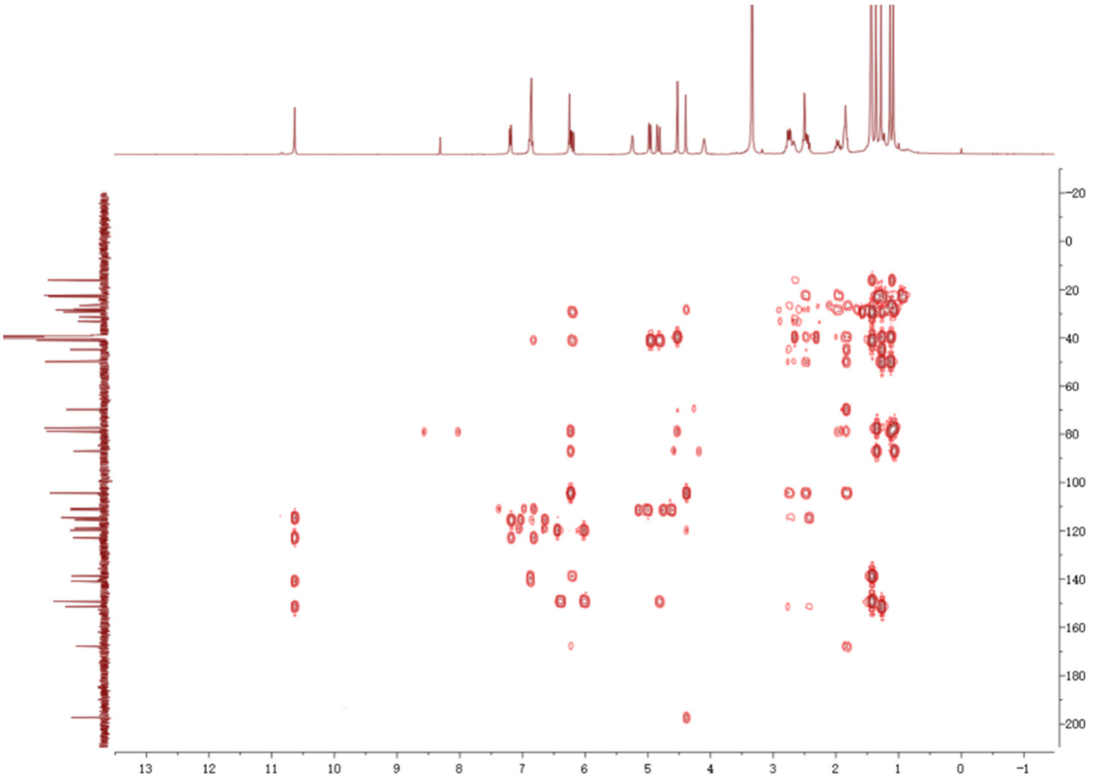

Figure S6. HMBC spectrum of **1** in DMSO-d_6

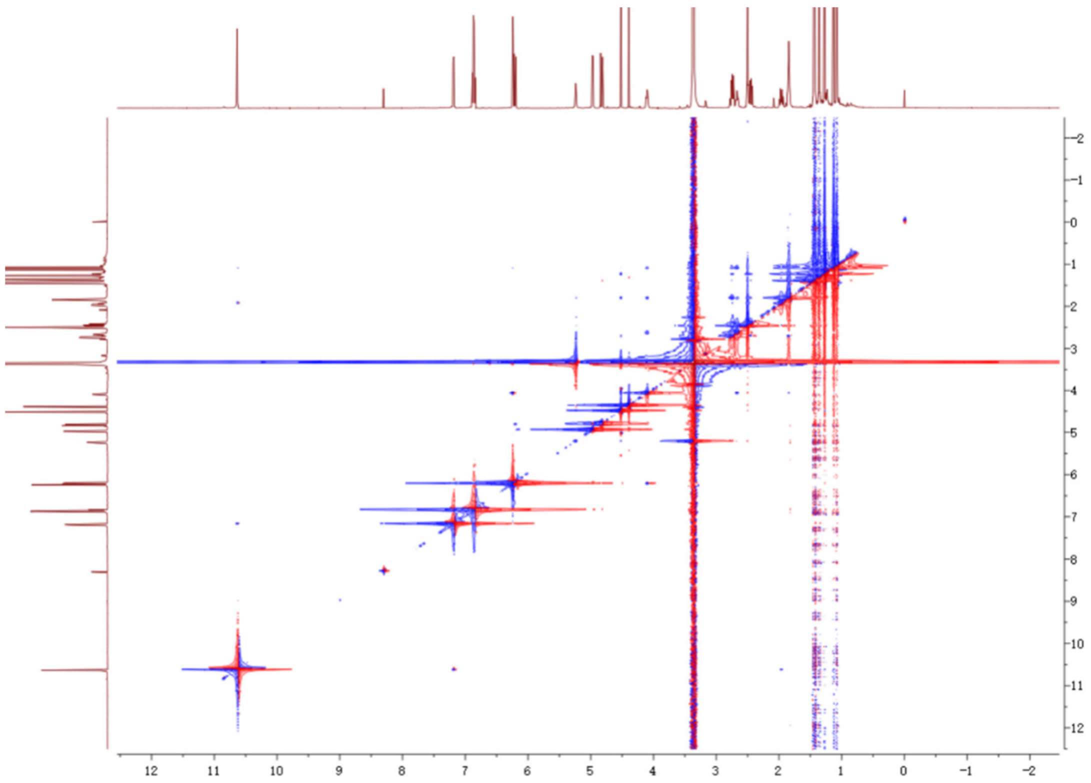

Figure S7. NOESY spectrum of **1** in DMSO-d_6

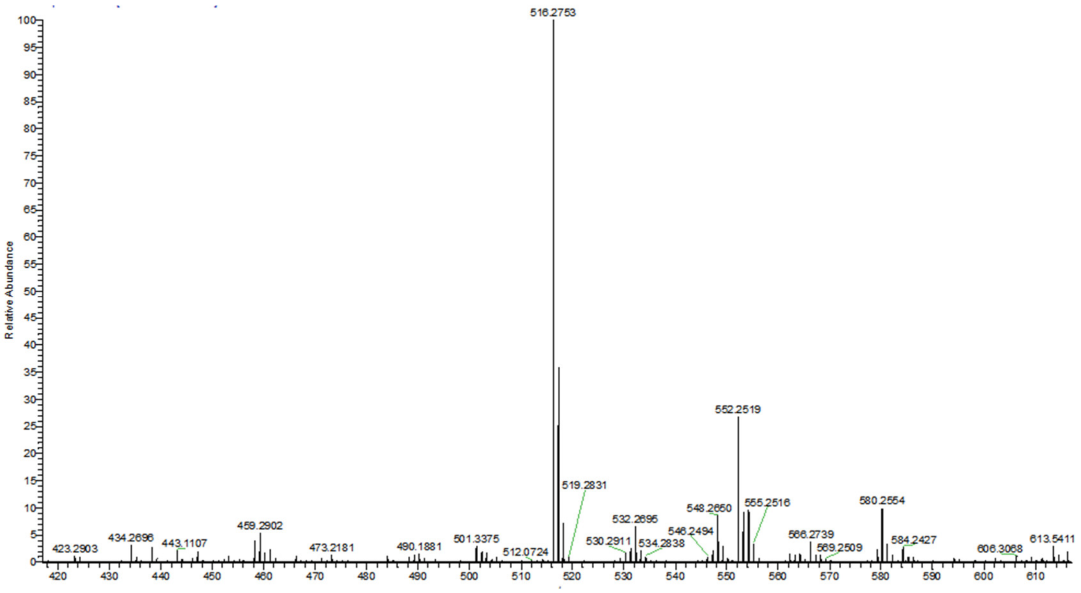

Figure S8. HRESIMS spectrum of **1**

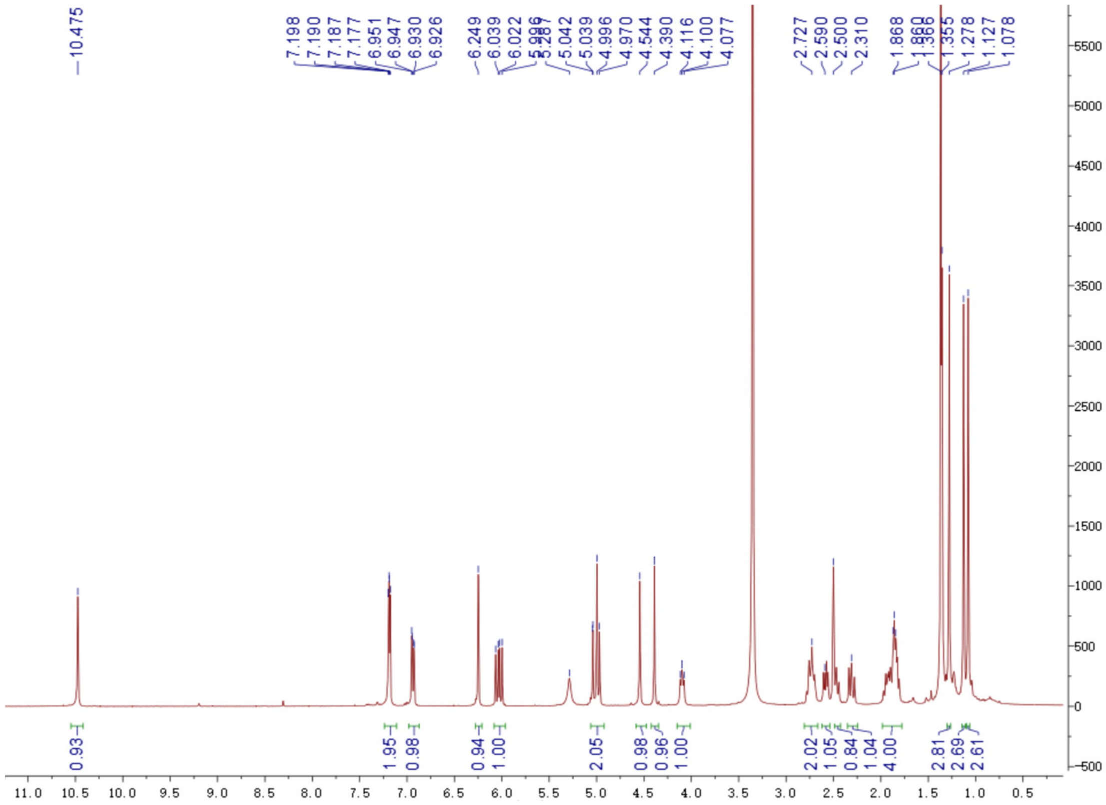

Figure S9. ^{1}H NMR spectrum of **2** in DMSO-d_6

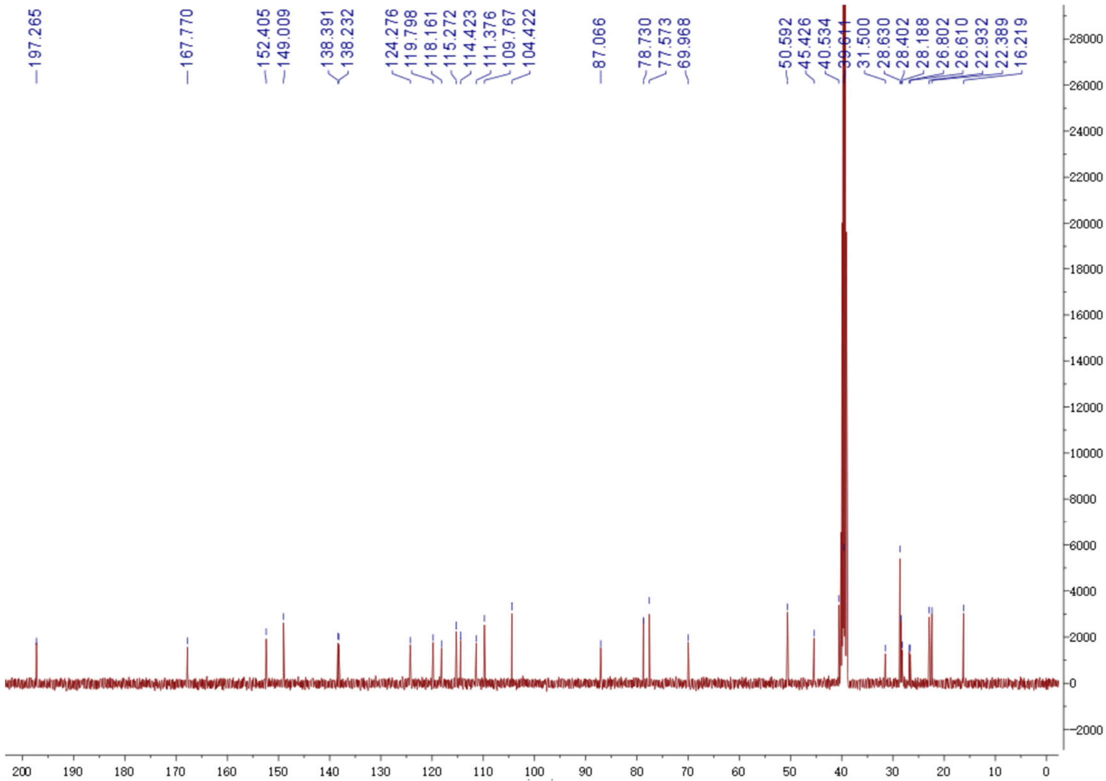

Figure S10. ^{13}C NMR spectrum of **2** in DMSO-d_6

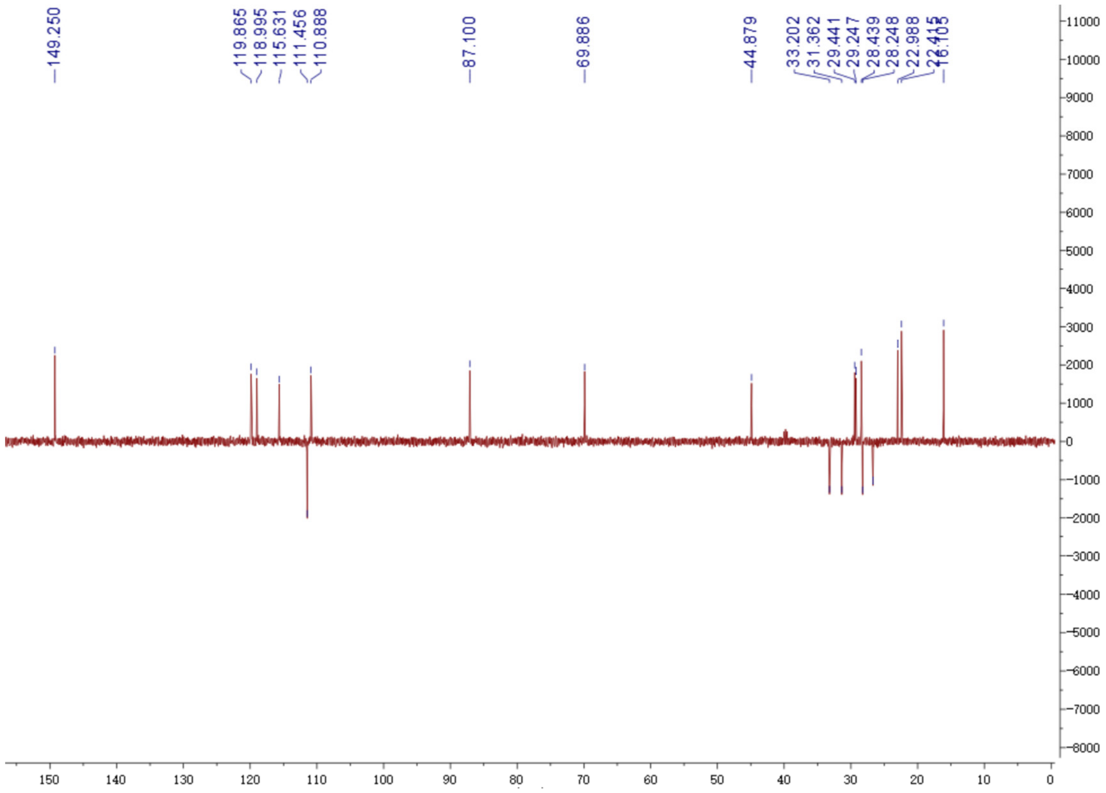

Figure S11. 135-DEPT spectrum of **2** in DMSO-*d*6

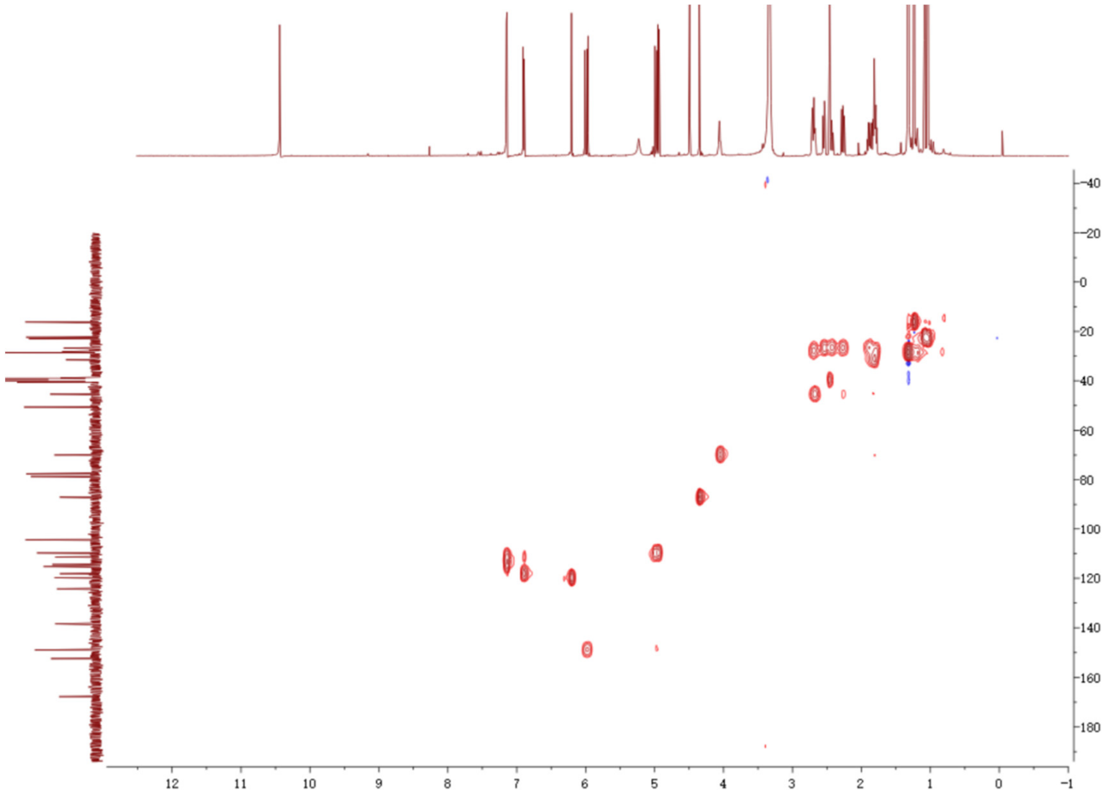

Figure S12. HSQC spectrum of **2** in DMSO-*d*6

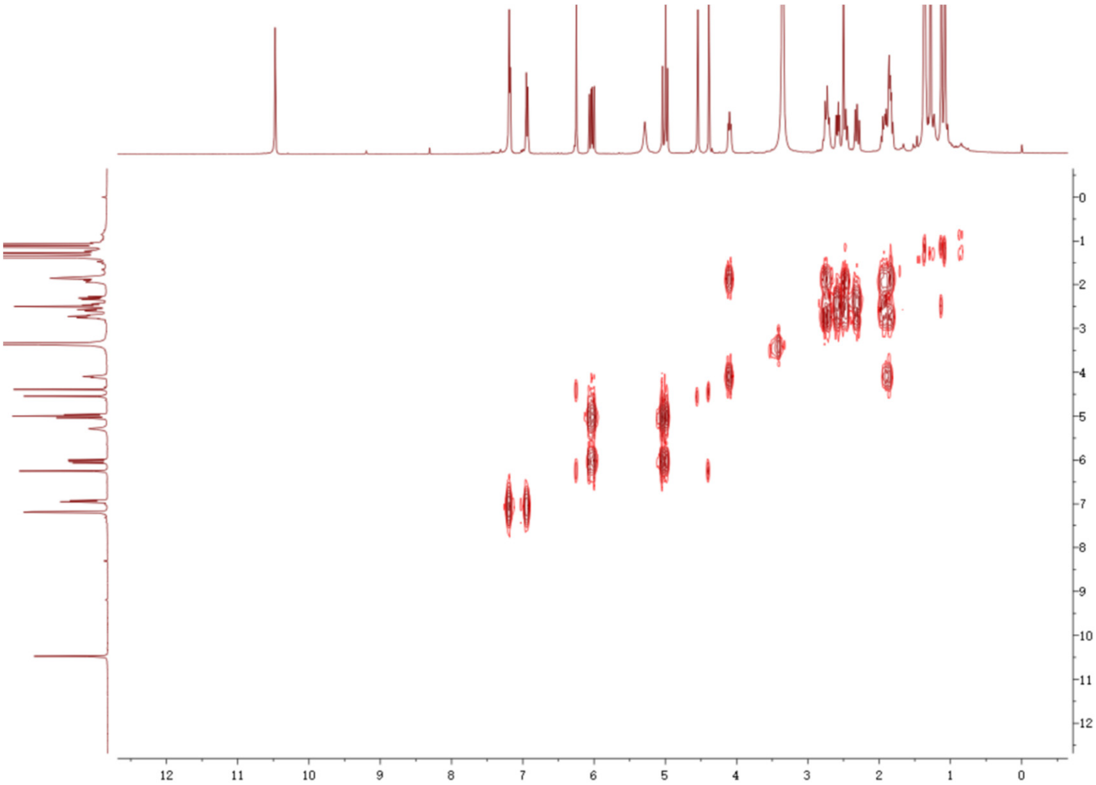

Figure S13. ^{1}H-^{1}H COSY spectrum of **2** in DMSO-*d*6

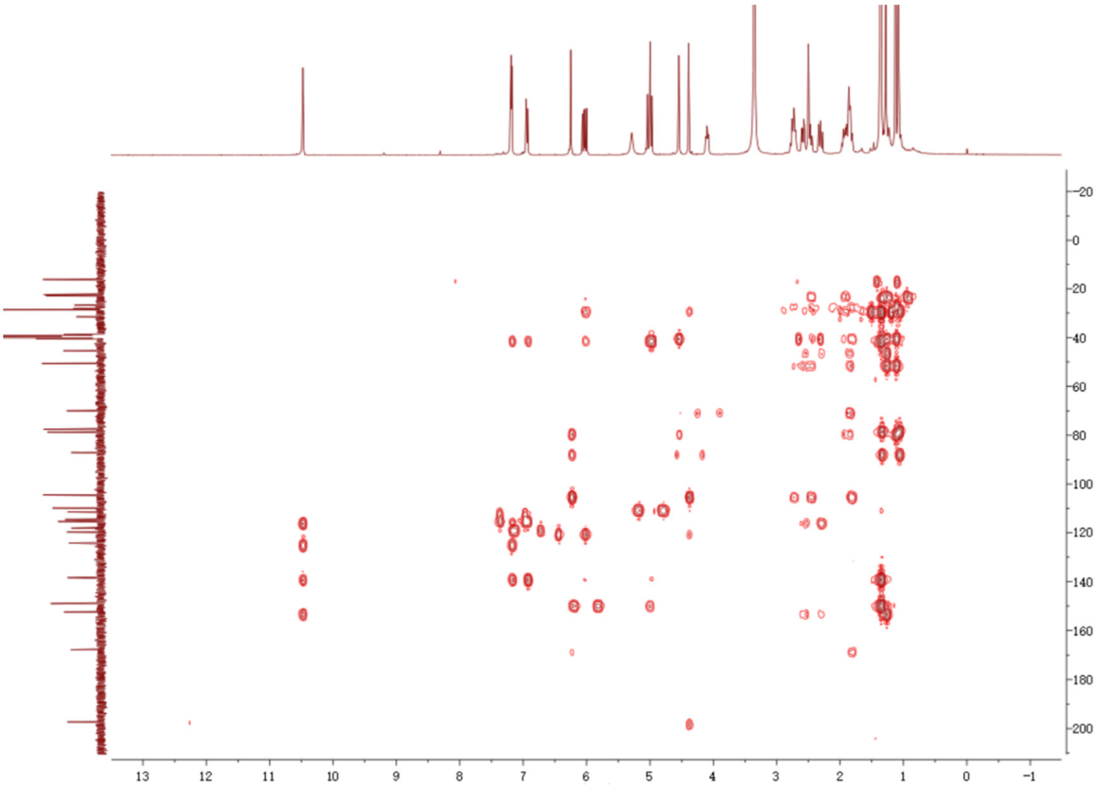

Figure S14. HMBC spectrum of **2** in DMSO-*d*6

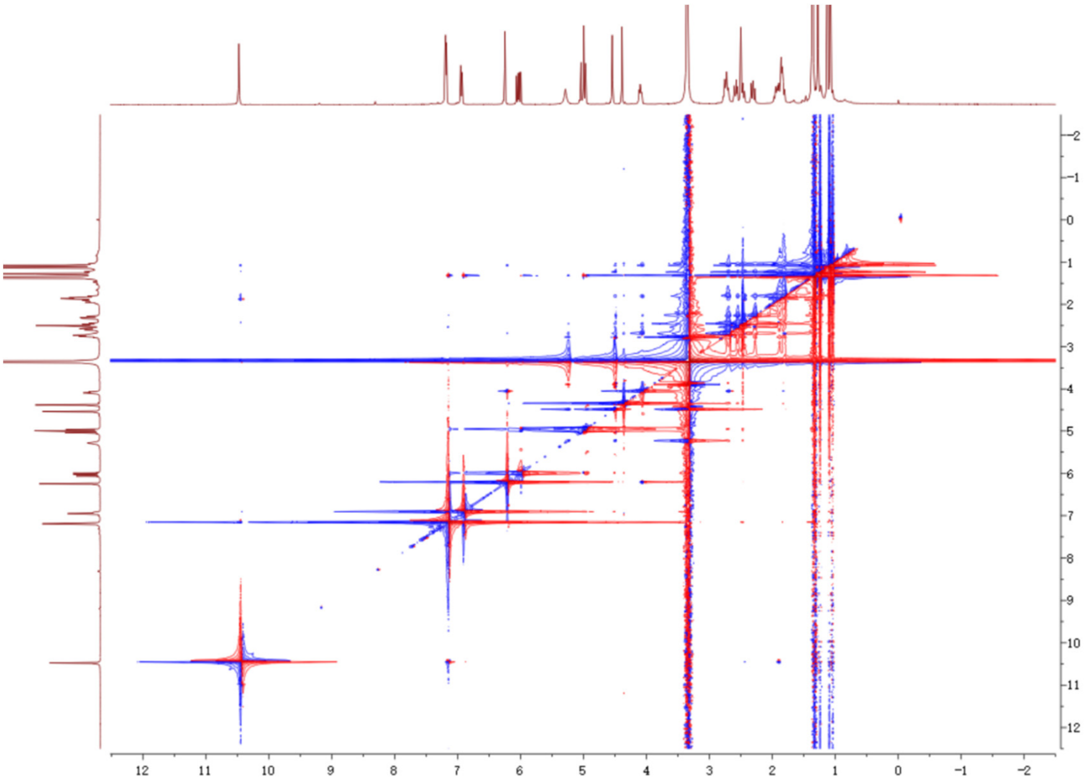

Figure S15. NOESY spectrum of **2** in DMSO-d_6

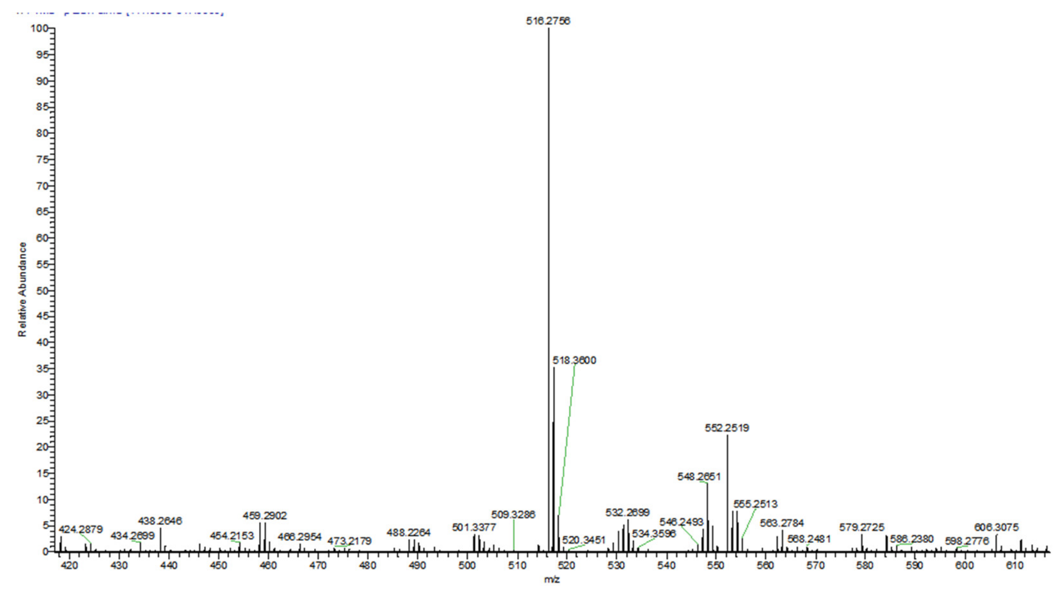

Figure S16. HRESIMS spectrum of **2**
